# Office-Based Laryngeal Biopsy in Patients Ineligible for General Anesthesia

**DOI:** 10.22038/ijorl.2020.42544.2436

**Published:** 2020-11

**Authors:** Francesco Mozzanica, Francesco Ottaviani, Daniela Ginocchio, Antonio Schindler

**Affiliations:** 1 *Department of Clinical Sciences and Community Health, University of Milan, Milan, Italy and Department of Otorhinolaryngology, Ospedale San Giuseppe IRCCS Multimedica, Milan, Italy.*; 2 *IRCCS Santa Maria Nascente – Fondazione Don Gnocchi, Milan, Italy.*; 3 *Department of Biomedical and Clinical Sciences, L. Sacco Hospital, University of Milan, Milan, Italy.*

**Keywords:** Biopsy, In-office procedure Larynx, Local anesthesia

## Abstract

**Introduction:**

Office-based laryngeal biopsy (OBLB) may provide a histological examination of laryngeal lesions in patients who cannot undergo a direct laryngoscopy. Nonetheless, only scarce information regarding its clinical applicability in these patients are available. The study’s aim is to report the feasibility of OBLB in patients ineligible for direct laryngoscopy.

**Materials and Methods:**

A total of 55 patients presenting with laryngeal lesions requiring biopsy but ineligible for direct laryngoscopy because at risk for general anesthesia were consecutively enrolled. OBLB was performed using a flexible endoscope with a 2 mm instrument channel under local anesthesia on an outpatient basis. The biopsied lesions were categorized according to their location, morphology, and histology (benign, premalignant, and malignant). In case of malignancy the patients started non-surgical treatment; otherwise, the patients were scheduled for a close follow-up.

**Results:**

OBLB was well tolerated and no complications occurred. Laryngeal lesions were more frequently located in the glottic region (28 out of 55 patients), while the most frequent morphology was ulcerative (35 out of 55 patients). The histological examination revealed 34 cases of malignancy, 9 cases of premalignancy, and 12 cases of benign lesions. In none of the patients without malignancy the laryngeal lesion showed significant changes during the follow-up period and a re-biopsy was not performed.

**Conclusion:**

In patients ineligible for direct laryngoscopy under general anesthesia OBLB could be considered as a sound-alternative method to assess the histology of suspected laryngeal lesions.

## Introduction

Office-based laryngeal surgery was performed routinely in surgeons’ offices in the nineteenth century. However, because of the advent of safe techniques for direct laryngoscopic surgery under general anesthesia and of precision instruments (such as the operating microscope and the CO2 laser), the majority of laryngeal surgeries were moved to the operating room (1). In this last decade, in order to develop less invasive and cost-efficient health-care methods for both diagnosis and treatment of laryngologic diseases, the interest in indirect laryngeal procedures renewed (2). The advantages of this approach are noteworthy. First of all, the surgeon is able to evaluate the voice quality during the procedure because the patient is awake and able to phonate. Second, office-based laryngeal surgery decreases patient cost compared to the operating room (3-5). Finally, it avoids the complications related to general anesthesia and rigid instrumentation (6).

The expand of in-office laryngeal procedures is related to several factors. Chief among these factors are the technological advances. In particular the introduction of high-definition distal-chip nasopharyngoscopes have allowed clinicians to obtain excellent image quality, and side channels that allows air insufflation, suction and the introduction of small flexible forceps, needles and laser fibers (6-10). Probably in certain aspects of laryngology, the advantages of direct laryngoscopic surgery, such as bimanual dexterity, high-powered magnification and a still operating field, cannot be replaced. However, the office-based indirect laryngeal surgery appears to be a sound-alternative approach for some laryngologic diseases. Several authors (11-16) reported their experience in the treatment of leukoplakia, Reinke’s edema, vascular ectasias, granuloma, vocal fold polyps, stenosis and papilloma using an in-office laser treatment. Moreover, also in-office vocal fold injection has been described (4,10). 

Finally, thanks to the flexible endoscopes with an operating channel, also the awake biopsy of the laryngopharyngeal region has become easier to perform and better tolerated by patients, founding its indications for lesions with difficult exposure and for patients with high risk related to general anesthesia (6). Even if several authors suggested that the office-based laryngeal biopsy (OBLB) represents a safe, reliable, and cost- and time- effective procedure (17-19), its application in the clinical practice is debated. Cha et al. (19) recommended to verify the results of OBLB with operative laryngeal biopsy performed under general anesthesia (ORLB) when severe dysplasia or carcinoma in situ (CIS) are reported in OBLB, or when the lesions are clinically suspicious for malignancy. Cohen et al. (18) recommended the verification with ORLB also in case the results of OBLB are reported as premalignant or benign lesions (18, 19). Richards et al. (20) concluded that OBLB could represent a valuable alternative to ORLB for benign lesions, but might be inappropriate to screen for malignancy because of its low sensitivity (60%). On the contrary, Castillo Farias et al. (21) reported that the sensitivity of OBLB for malignancy reaches the 81.1% and suggested its application as an initial diagnostic modality for laryngopharyngeal malignancy (19), while Lippert et al. (17) recommended the routine use of OBLB because it leads to an earlier treatment (19).

These diverging results could be related to several factors such as: different number of enrolled patients, or differences in: criteria used for patients’ selection, study design (prospective/retrospective), and length of follow-up period after OBLB (19). Thus, the routine use of OBLB is still controversial and no concrete algorithm for its clinical application has been proposed so far. Nonetheless, OBLB may represent the only alternative to ORLB in patients who cannot undergo a direct laryngoscopy because not eligible for general anesthesia (for example for cardiologic, neurologic, pneumological causes) or because patients’ anatomical characteristics prevent a satisfactory laryngeal exposure (for example for macroglossia, limited extension of the cervical spine, obesity, difficulties in opening the mouth, inflexible necks, and retrognathia (22)). For this reason, additional information regarding the clinical applicability of OBLB as a diagnostic tool for laryngeal lesions are needed. In the present study our experience on 55 patients with suspected laryngeal lesions who underwent OBLB because ineligible for general anesthesia is reported. The underlying hypothesis is that OBLB is a safe, specific and sensitive diagnostic tool for laryngeal lesions and could be used as a diagnostic procedure in patients who cannot undergo direct laryngoscopy. 

## Materials and Methods

**Participants**

A total of fifty-five patients (5 females and 50 males) presenting with laryngeal lesions requiring biopsy were consecutively enrolled in the period between January 2010 and January 2016. The mean age of the cohort was 67 ± 6.7 years (range 55-82 years). Inclusion criteria were: age above 18 years, presence of laryngeal lesions visible on office endoscopy, ineligibility for direct laryngoscopy. None of the enrolled patient had an history of head and neck malignancy. This study was carried out according to the Declaration of Helsinki and was previously approved by Institutional Review Board of our hospital.

**Surgical procedure**

All the enrolled patients underwent OBLB using a flexible endoscope with a 2 mm instrument channel connected to a video processor with a 100-W xenon light source. The outer diameter of the endoscope was 4 mm; reusable fenestrated round-cup biopsy forceps were used for all the biopsies. 

**Fig1:**
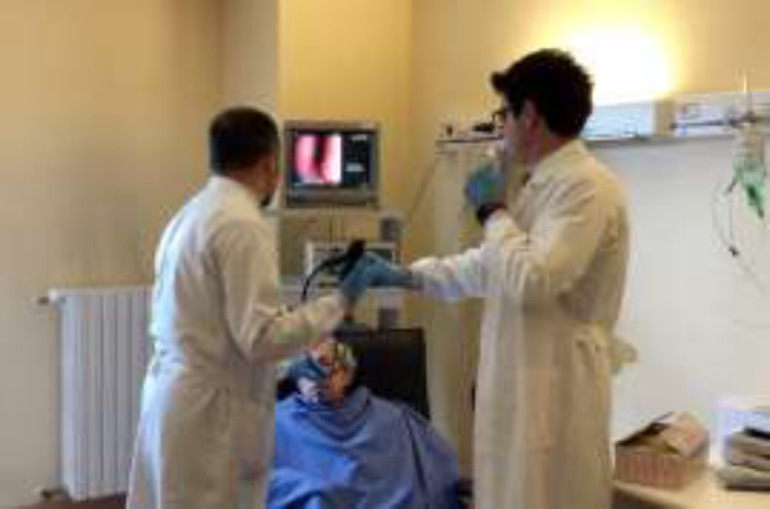
Picture showing the position of the patient and of the two surgeons during the office-based laryngeal biopsy (OBLB). The patient is in a semi-seated position, the first surgeon maneuvers the endoscope, while the other one maneuvers the biopsy forceps

The procedure was performed under local anesthesia on an outpatient basis after formal written consent was obtained. All the procedures were performed by two surgeons: one maneuvered the endoscope and the other one maneuvered the biopsy forceps (Fig.1). 

**Fig2:**
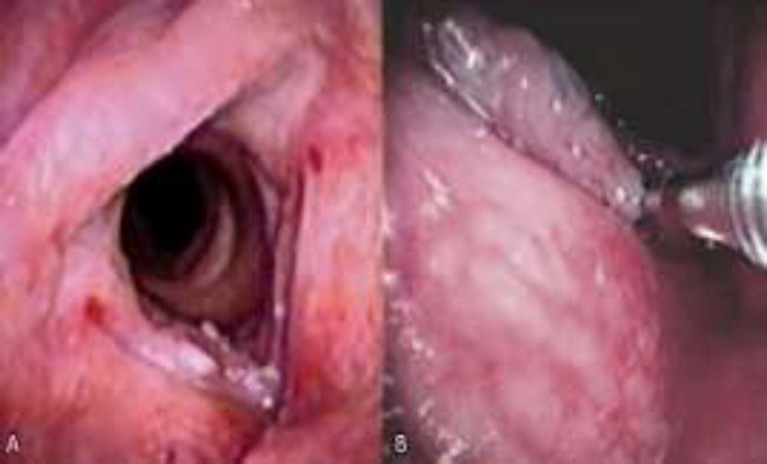
Scope view at the time of tissue sampling using office-based laryngeal biopsy (OBLB): A: pre-operative view of the laryngeal lesions; B: the forceps are inserted through the working channel of the operative flexible endoscope and the lesions are biopsied using the forceps

The examiner clearly explained the whole procedure to the patient, whose active collaboration is necessary. Patients were put in a semi-seated position, local anesthesia was provided into the pharynx and nose using 10% lidocaine. In addition, in order to improve the visualization of the larynx the head of the patient was tilted backwards. Once the endoscope was inserted, the larynx was anesthetized by instillation of lidocaine (first at 2% and then at 10%) through the endoscope’s working channel. The lesion was then brought out through the nose while the endoscope was simultaneously withdrawn (Fig.2). Immediately afterwards the occurrence of complications was checked through an endoscopic examination. After the OBLB, patients were asked to avoid eating and drinking in order to reduce the risk of aspiration secondary to the topical anesthesia.

**Lesions’ categorization**

Laryngeal lesions were categorized on the basis of their morphology (plaque, elevated, fungating, and ulcerative), location (subglottic, glottic and supraglottic), and histological findings. In particular, similar to the study of Cha et al. (19) the lesions were distinguished among malignant (for example squamous cell carcinoma or lymphoma), premalignant (CIS, severe, moderate or mild dysplasia) and benign (acanthosis, papilloma, reactive lesion, inflammation, and keratosis).

**Post-operative evaluation and follow-up**

The type and number of complications occurred during or after OBLB were analyzed. In case of malignancy the patients were referred to a multidisciplinary oncology consultation in order to start treatment. When no malignancy was found at the initial OBLB, the patients were scheduled for a close follow-up for at least 42 months (mean 54 12 months, range 42-96 months) in order to rule out malignancy. During the follow-up visit a laryngoscopic examination using a flexible or rigid endoscope was performed. OBLB was performed only in case of a significant change in the lesion’s characteristics demonstrated during the follow-up laryngoscopic examination. 

## Results

All the 55 patients enrolled in the study underwent OBLB because ineligible for general anesthesia due to cardiologic (29 patients), pneumological (19 patients), or neurologic diseases (7 patients). The time between the initial evaluation and the OBLB never exceeded 5 working days (mean 2.9 ± 2.1 days). 

The procedure was well tolerated and no complications, such as bleeding, occurred. The procedure did not take more than 30 minutes to be completed, including the application of local anesthesia. During each procedure a mean of 2.4 biopsies were executed (range 2-6). The number of biopsies was mainly related to the size of the lesion. In all the patients the obtained tissue was sufficient for histopathologic diagnosis. As far as it concerns the location of the suspected lesions, in 28 cases (50.9%) it was the glottic region, in 23 cases (41.8%) it was the supraglottic region, while in 4 cases (7.3%) it was the subglottic region. The most frequent morphology was ulcerative (35 patients, 63.6%), followed by plaque (10 patients, 18.2%). The elevated and fungating morphology were less common (7 patients, 12.7%, and 3 patients, 5.5%, respectively). 

 The histological examination of the biopsied lesions revealed 34 cases of malignancy (61.8%, squamous cell carcinoma in all the cases), 9 cases (16.4%) of premalignancy (mild dysplasia in 6 and moderate dysplasia in 3 cases), and 12 cases of benign lesions (21.8%, 8 cases of keratosis, 3 cases of papilloma and 1 case of acanthosis). 

All the 34 patients with a malignancy at OBLB were referred to the multidisciplinary oncology consultation in order to start non-surgical treatment. A combination of chemo- and/or radiotherapy was performed since none of these patients was eligible for general anesthesia due to the high risk of this procedure. Radiation therapy was the commonest modality of treatment (30 patients, 88.2%), followed by chemoradiotherapy (4 patients,11.8%). In the remaining 21 patients the OBLB did not demonstrated malignancy. Consequently, they were scheduled for a close follow-up with videolaryngoscopic examination every 2 months for at least 3 years and then every 6 months. In none of these patients the laryngeal lesion showed significant changes during the follow-up period and a re-biopsy was not performed. 

## Discussion

In the present study our experience with OBLB performed in patients with suspected laryngeal lesions and ineligible for direct laryngoscopy is presented. The results here reported appear interesting. First of all, no post-operative complications were reported. In particular, in none of the enrolled patient vasovagal reaction, post-procedure aspiration, epistaxis, or bleeding from the biopsied lesion were demonstrated (23). This finding agrees with those of Cohen et al. (24) who analyzed the adverse events in OBLB in a cohort of 390 patients and reported a very low level of complications all of which were mild (epistaxis in 2 patients, hematoma of the vocal fold in 1 patient, and aspiration in 1 patient). Similarly, also other previous studies confirmed that patients experience minimal to no complications from OBLB (17,18,25-27). The absence of complications reported in our study might be related to the relatively small number of enrolled patients. However, it is possible to speculate that an adequate patient’s preparation might have played a role. For example, our patients were instructed to avoid eating and drinking after the OBLB and this might have reduced the risk of aspiration events. In addition, an endoscopic examination was performed immediately after OBLB in order to verify that no complication occurred. In case of complications, such as bleeding, this could have allowed their prompt identification and treatment. 

Similar to previous reports (23), the OBLB was well tolerated in all the enrolled patients. It is possible to speculate that the satisfactory feasibility of OBLB depends on the use of local anesthetic and on the use of instruments inserted using the endoscope’s operating channel (which reduced the discomfort). The good tolerability of OBLB might explain the high rate of adequate sampling (100%) found in the present study. Similar findings were reported by Cha et al. (19) and are probably related to the fact that in our series more than 1 biopsy for each patient was performed. 

OBLB seems to offer a very short diagnostic workup time since the delay from initial consultation to biopsy never exceeded 5 working days (mean 2.9 days). This datum might be related to the higher availability and cost-effectiveness of OBLB compared to biopsies performed using direct laryngoscopy and general anesthesia (24). Lee et al. (6) who compared the time to diagnosis in patients undergoing OBLB or ORLB for laryngopharyngeal lesions, found that patients in the OBLB group received a tissue biopsy 1.3 days after the initial consultation. Also, Cohen et al. (24) found that in the majority of patients with suspected laryngeal lesions the OBLB offered a reduction in diagnostic workup time, while Lippert et al. (17) reported that a successful in-office biopsy assured an average time saving of 24.6 days to treatment. The reduced diagnostic evaluation period offered by OBLB should be considered one of the most important advantages of this procedure. By speeding up the patient’s diagnostic workup time, in fact, OBLB might also diminish the period to the treatment (24). 

Despite the advantages, several authors express concerns regarding the diagnostic clarity of OBLB (6). In particular, the sensibility of this procedure largely varies across previous studies, ranging from 60% in the study of Richards et al. (20), to 81.1% in the study of Castillo Farias et al. (21). This relatively low level of sensibility limits the clinical applicability of OBLB as a diagnostic tool. For this reason, several authors suggested to perform a confirmation biopsy using ORBL (18,19,24). In our sample a confirmation biopsy was not performed because the patients were ineligible for ORBL because at high risk for general anesthesia. Only one previous study analyzed the efficacy of videolaryngoscopic surgery in patients who were not suitable for phonosurgery by microlaryngoscopy (28). The authors reported a high rate of success rate when treating laryngeal polyps (95.5%), Reinke’s edema (89%) and cysts of the vocal folds (52.3%), while in cases of suspicious lesions the OBLB allowed a diagnosis in all cases (28). 

This study has several limitations. First of all, confirmatory biopsy using ORLB was not performed. Consequently, no information regarding the sensibility, specificity, positive and negative predictive valued of this procedure are reported. For this reason, information regarding the clinical utility of OBLB may only be inferred. In our sample all the 34 patients with malignancy were submitted for further non-surgical treatments. The remaining patients were scheduled for a close follow-up for at least 42 months. In none of these patients the laryngeal lesion showed significant changes during the follow-up and a re-biopsy was not performed, thus suggesting that the initial diagnosis performed through OBLB was correct. A second limitation lies in the limited number of enrolled patients. Consequently, the results here reported should be considered as preliminary. However, it must be noted that the small number of enrolled patients is mainly related to the fact that only patients affected by suspected laryngeal lesions who were not suitable for direct laryngoscopy under general anesthesia were enrolled. 

## Conclusion

OBLB is a simple, safe, and minimally invasive procedure for laryngeal biopsy even in patients who cannot undergo general anesthesia. The patient’s active collaboration is minimal thanks to the efficacy of the topical anesthesia and to the trans nasal approach. In addition, OBLB offers a fast-diagnostic workup but its sensibility is still a matter of debate. Even if is true that ORBL represents the gold standard in the diagnosis of laryngeal lesion, in patients ineligible for direct laryngoscopy under general anesthesia OBLB could be considered as a sound-alternative method to assess the histology of suspected laryngeal lesions.
